# A Case of Herpes Zoster Ophthalmicus in a Recently Transplanted Renal Patient

**DOI:** 10.7759/cureus.40899

**Published:** 2023-06-24

**Authors:** Mohamad Jabin, Zaryab Alam, Evan Chung, Mojahed Shalabi, Bismah Siddiqui

**Affiliations:** 1 Medicine, Texas A&M College of Medicine, Bryan, USA; 2 Medicine, Burnett School of Medicine, Fort Worth, USA; 3 Internal Medicine, Baylor Scott & White All Saints, Fort Worth, USA

**Keywords:** herpes zoster ophthalmicus, shingle skin rash, shingles complications, trigeminal nerve, varicella-zoster virus

## Abstract

Herpes Zoster Ophthalmicus (HZO) is a common manifestation of the reactivated Varicella Zoster virus, primarily affecting the eye and trigeminal nerve. This case study presents the clinical course of a 51-year-old male who underwent a renal transplant due to end-stage renal disease, further complicating the management of HZO. The patient's medical history also includes hypertension, type 2 diabetes mellitus, chronic kidney disease (CKD), cerebrovascular accident (CVA), and retinal detachment. Upon examination, the diagnosis of HZO was confirmed based on the presence of a characteristic unilateral vesicular rash in the V1 cranial nerve dermatomal distribution, accompanied by ophthalmic symptoms such as eyelid swelling and visual impairment. Given the patient's immunosuppressive regimen post-transplant, intravenous acyclovir was initiated for antiviral therapy, while supportive care was provided for pain control. Notably, the patient experienced a subsequent decrease in pain intensity and improvement in the vesicular rash. This case highlights the challenges in managing HZO in patients with a history of renal transplant and multiple comorbidities, emphasizing the importance of tailored treatment strategies to optimize patient outcomes. Further research is warranted to better understand the impact of immunosuppression and comorbidities on the course and management of HZO in this population.

## Introduction

Herpes Zoster Ophthalmicus (HZO) affects around 15% of individuals with herpes zoster [1]. HZO is caused by the reemergence of the varicella-zoster virus within the trigeminal ganglion, presenting as a rash with vesicles on an erythematous base accompanied by ophthalmalgia. HZO can induce ocular symptoms such as keratitis, uveitis, retinal perivasculitis, and necrosis. It may result in permanent vision loss in severe cases. The risk of developing HZO increases with age and other factors, such as solid organ transplant. Diagnosis is made through physical examination, medical history, and pathognomonic findings. Treatment involves antiviral therapy and corticosteroids for nerve inflammation and should be started within 72 hours of onset to be effective at preventing long-term effects.

## Case presentation

**Figure 1:**
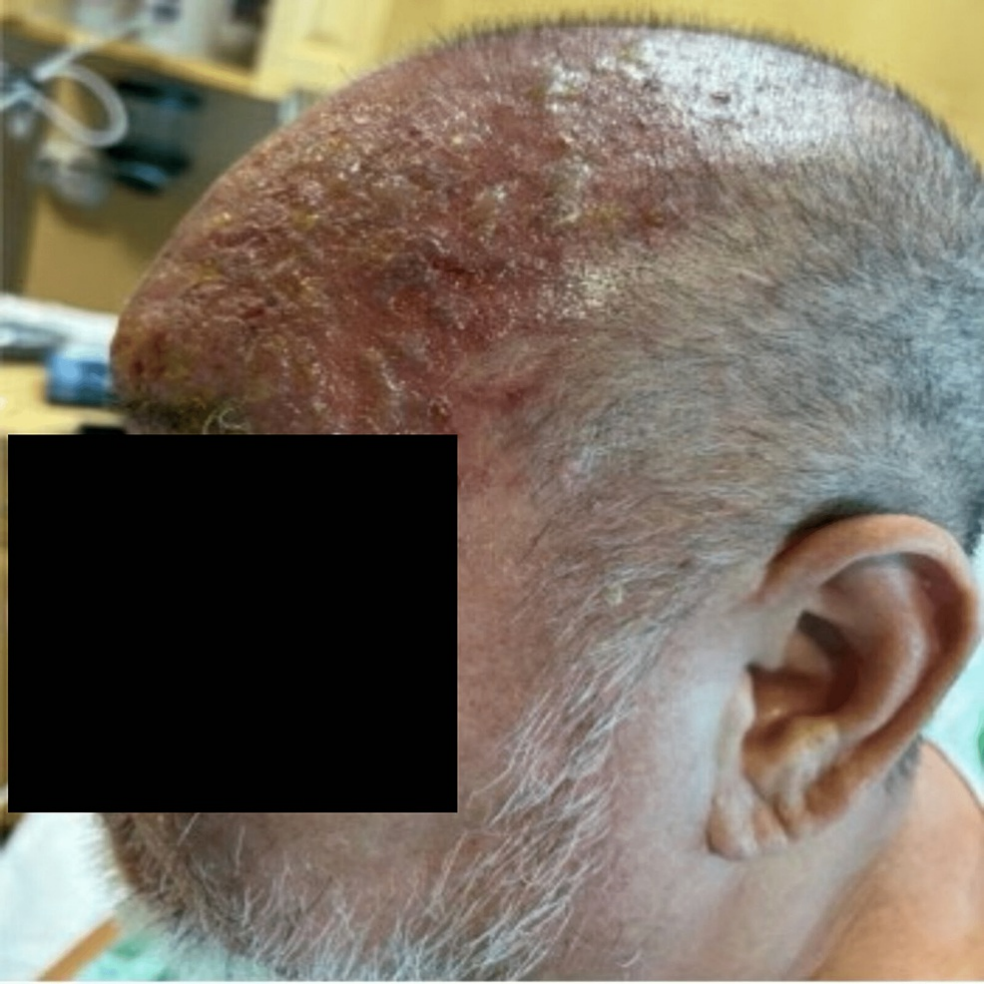
Erythematous, vesicular rash in a CN V1 distribution that is secondarily impetiginized with associated blepharitis.

**Figure 2:**
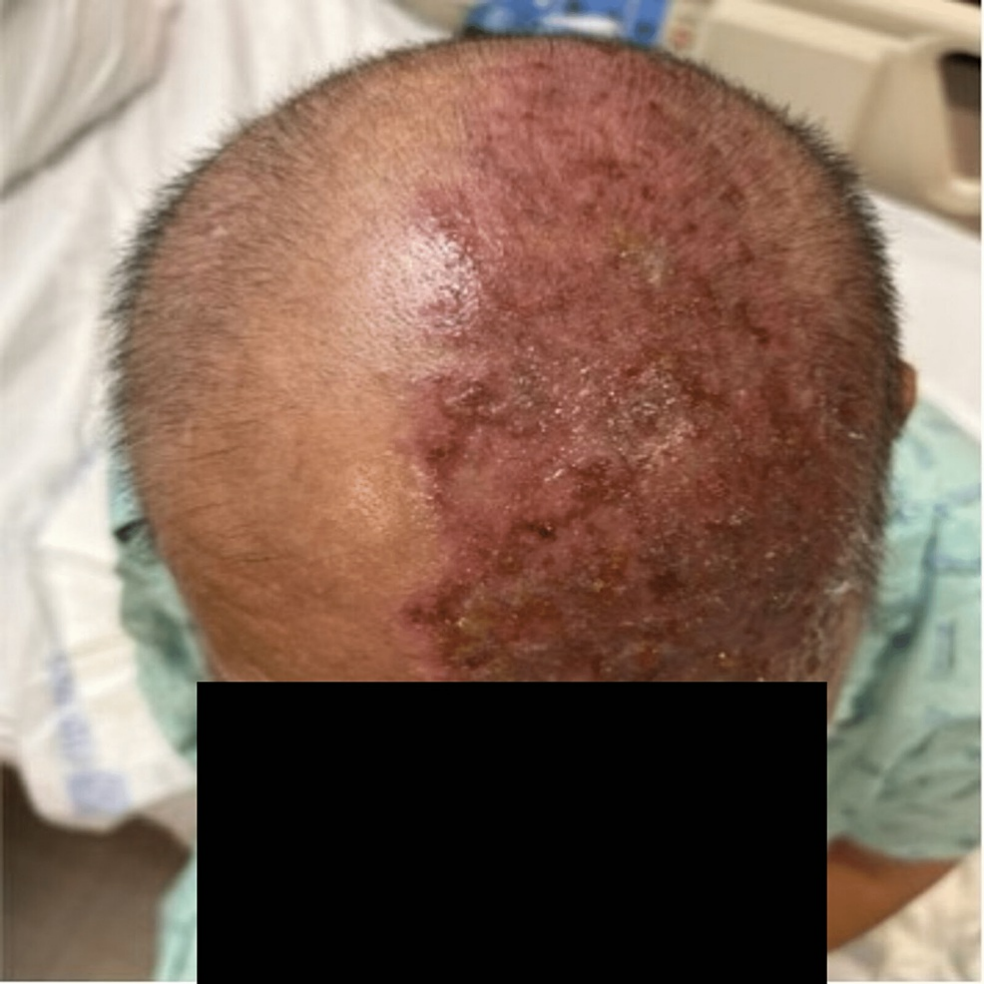
Well demarcated, erythematous, and vesicular rash on the scalp with hemorrhagic crust.

**Figure 3:**
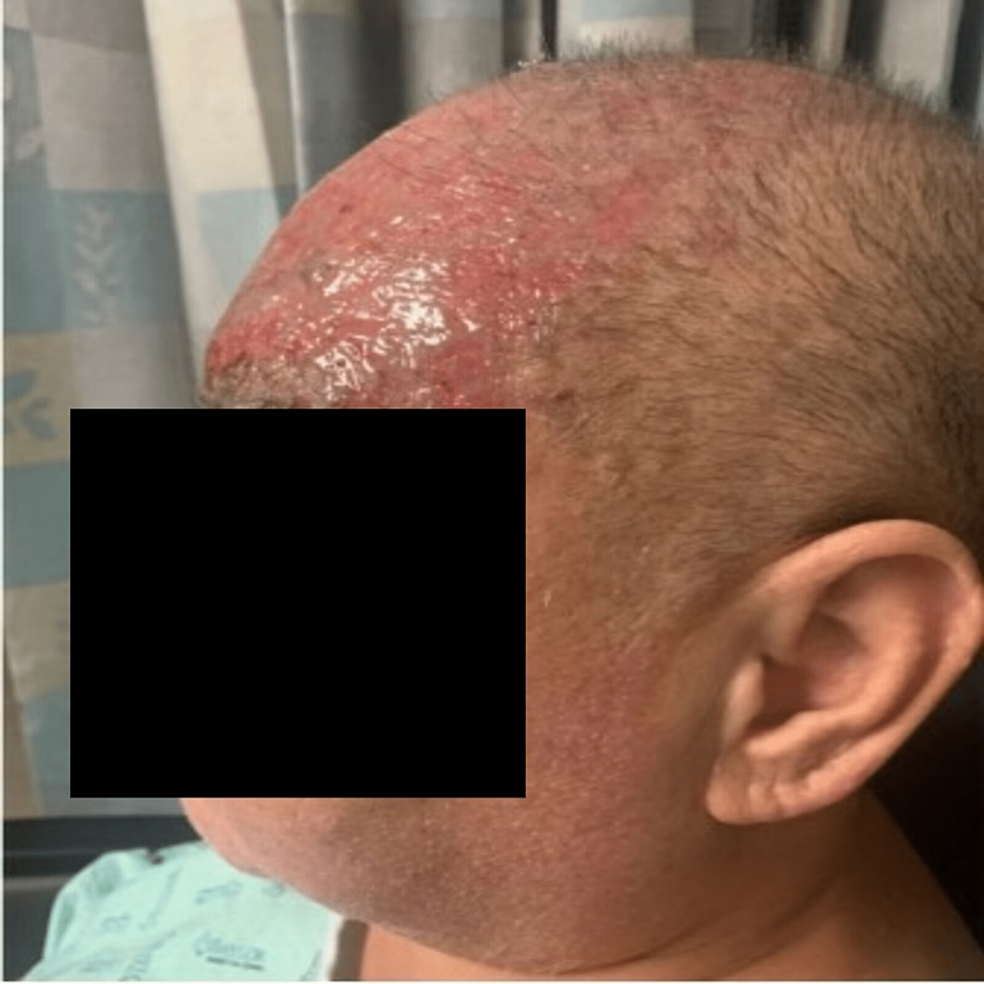
Improvement of the rash with evidence of fading erythema after 5 days of Acyclovir.

**Figure 4:**
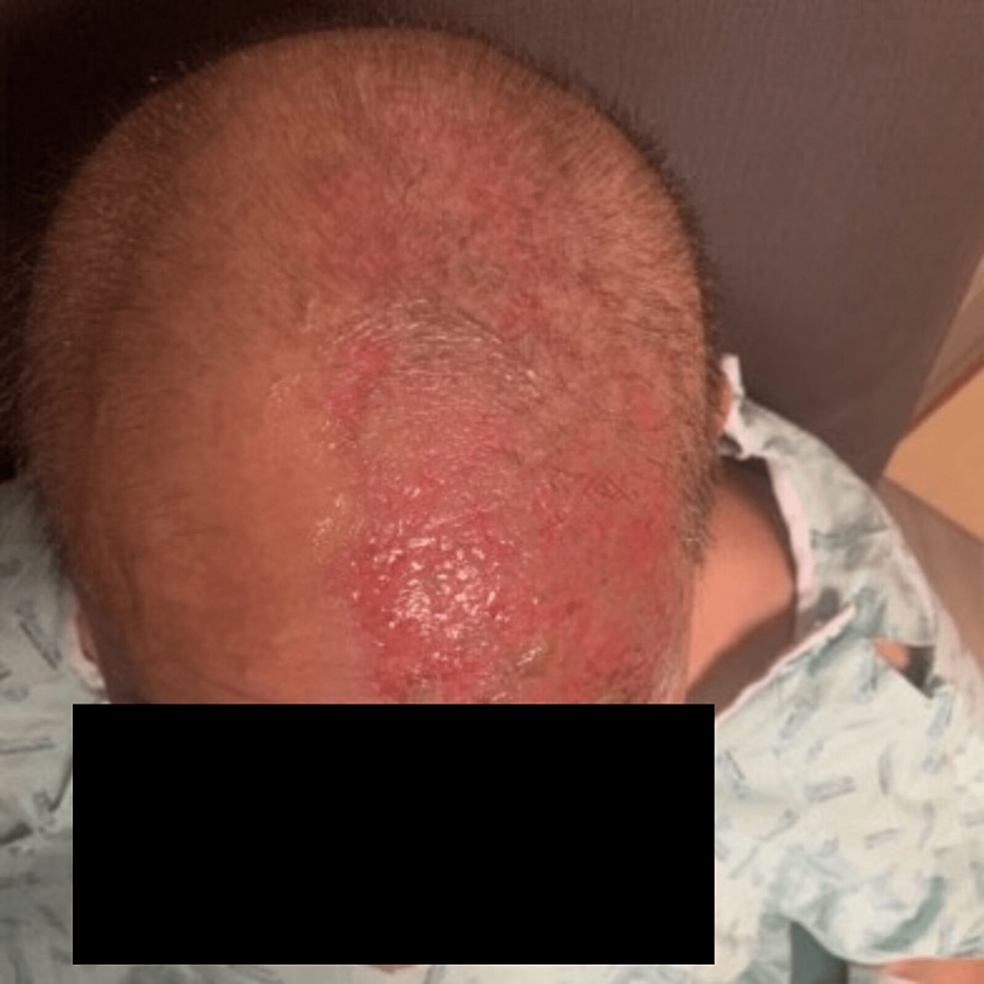
Improvement of the rash with evidence of fading erythema after 5 days of Acyclovir.

A 51-year-old male presented to the emergency department with a unilateral, vesicular rash on the left side of his face that extended from the crown of the head to the left eyelid with impetiginization of the vesicles in his scalp and forehead, in a V1 cranial nerve dermatomal distribution (Figure 1, Figure 2). The patient experienced localized aching pain in the same region a day prior to the rash, which was not responsive to acetaminophen. The patient experienced a concurrent rash along with a progressively worsening constant, burning pain rated at 9 out of 10 on the numeric pain scale. The patient denied other symptoms. His past medical history included type 2 diabetes, chronic kidney disease (CKD), cerebrovascular accident (CVA), and a renal transplant due to end-stage renal disease. On a neurological examination, he had normal muscle strength and was alert and oriented to person, place, and time. His left eye was swollen shut and the left eyelid erythematous. He was noted to be legally blind in his left eye. He was started on IV acyclovir for herpes zoster ophthalmicus and received prophylactic treatment with IV vancomycin and Zosyn. The patient reported decreasing pain day by day, with the pain completely gone by Day 5. The vesicular rash showed improvement daily, with increased crusting (Figure 3, Figure 4).

## Discussion

Herpes Zoster (HZ), commonly known as shingles, is caused by the reactivation of the Varicella Zoster virus, also known as human herpes virus 3 [2]. After a primary infection which results in chickenpox, the virus remains dormant in the dorsal root ganglion [3]. Reactivation of this virus ordinarily takes place in adulthood, with 68% of cases being in individuals over the age of 50, most likely due to a decline in cell-mediated immunity with age [4,5]. HZ has an incidence rate of 33% in the United States [6]. Once the virus has reactivated it manifests as a rash that follows a dermatomal pattern [7]. The rash consists of grouped vesicles that contain serous fluid on an erythematous base that eventually will crust over and heal [7]. A varicella vaccine has been a part of routine childhood vaccinations to prevent further complications of the virus, as well as a shingles vaccine offered to adults above the age of 50 [8]. The vaccines have been shown to not only decrease the risk of developing the disease but also decrease severity and complications even if the disease develops [8].

Roughly 15% of individuals infected with HZ will develop HZO, which is a reactivation of the Herpes virus in the ophthalmic branch of the trigeminal nerve [1]. Fifty percent of cases of HZO have ocular involvement with symptoms like, keratitis, uveitis, retinal perivasculitis and necrosis, optic neuritis, and scleritis [9]. One of the most debilitating complications of HZO is the development of permanent vision loss, which is seen in approximately 20% of patients [9].

This case of HZO in a recently transplanted renal patient is notable for multiple reasons. The patient's history of renal transplant and resulting immunosuppression adds complexity to managing HZO. The presence of multiple comorbidities, such as hypertension, type 2 diabetes mellitus, CKD, CVA, and retinal detachment, further complicates treatment. Additionally, the severity of the patient's pain, rated at 9 out of 10, highlights the significant impact of HZO on their quality of life and underscores the importance of effective pain management.

HZO is diagnosed primarily through a thorough history and physical examination. While diagnostic laboratory testing is rarely required, a Tzanck smear and PCR can be performed to detect the herpes virus from swabbing exudates of the lesion [10]. Due to the involvement of the ophthalmic branch of the trigeminal nerve, it is crucial to understand this nerve distribution, because of the reliance on a physical assessment for a diagnosis. The ophthalmic branch of the trigeminal nerve has three main divisions: the lacrimal, nasociliary, and frontal branches [11]. These branches are important because in 1864, Sir Jonathan Hutchinson discovered that if the HZ lesion involved the side, tip, or root of the nose, this physical exam finding is a powerful predictor that the patient will develop HZO [12]. This finding was later coined by the term Hutchinson’s sign. This sign has a relative risk factor of 3.35 (CI 95%:1.82-6.15) and 4.02 (CI 95%:1.55-10.42) of predicting ocular inflammation and corneal denervation respectively [13].

Early diagnosis and treatment of HZO by physicians can prevent the devastating sequela of this disease. Treating HZO is centered around antiviral therapy as well as corticosteroids for nerve inflammation management [14]. HZO treatment ideally should begin within 72 hours of disease onset, consisting of Acyclovir 800mg orally for a minimum of seven days and adjuvant therapy with either a topical or systemic corticosteroid for an immunocompetent patient [14]. Treatment for immunocompromised patients varies slightly with the dosage of Acyclovir being 10mg/kg administered IV [14]. A dosage of 90mg/kg IV of Foscarnet may be substituted for Acyclovir-resistant strains [14]. It has been shown that early treatment of HZO with Acyclovir within three days of onset has favorable effects on postherpetic neuralgia, as well as increases the speed of lesion resolution and decreases the incidence of uveitis and stromal keratitis [15,16].

## Conclusions

In conclusion, HZO is a common complication of the reactivated herpes zoster virus that significantly impacts the eye and trigeminal nerve. This case of HZO in a recently transplanted renal patient highlights the complexity and challenges associated with managing the disease in individuals with specific medical backgrounds and immunosuppressive regimens. Early diagnosis and prompt initiation of treatment are essential to prevent the potentially debilitating consequences of HZO. It is crucial for healthcare providers to recognize the importance of timely intervention and tailored management strategies to minimize the downstream effects of this disease. Further research and collaboration between specialties are warranted to enhance our understanding of HZO and develop effective evidence-based approaches to its diagnosis and treatment.
